# The Effect of Encapsulating a Prebiotic-Based Biopolymer Delivery System for Enhanced Probiotic Survival

**DOI:** 10.3390/polym15071752

**Published:** 2023-03-31

**Authors:** Aida Kistaubayeva, Malika Abdulzhanova, Sirina Zhantlessova, Irina Savitskaya, Tatyana Karpenyuk, Alla Goncharova, Yuriy Sinyavskiy

**Affiliations:** 1 Faculty of Biology and Biotechnology, Al-Farabi Kazakh National University, Almaty 050040, Kazakhstan; 2 Kazakh Academy of Nutrition, Almaty 050008, Kazakhstan

**Keywords:** coencapsulation, symbiotic beads, *Lactobacillus rhamnosus* GG, bacterial cellulose, pullulan, simulated gastrointestinal conditions

## Abstract

Orally delivered probiotics must survive transit through harsh environments during gastrointestinal (GI) digestion and be delivered and released into the target site. The aim of this work was to evaluate the survivability and delivery of gel-encapsulated *Lactobacillus rhamnosus* GG (LGG) to the colon. New hybrid symbiotic beads alginate/prebiotic pullulan/probiotic LGG were obtained by the extrusion method. The average size of the developed beads was 3401 µm (wet), 921 µm (dry) and the bacterial titer was 10^9^ CFU/g. The morphology of the beads was studied by a scanning electron microscope, demonstrating the structure of the bacterial cellulose shell and loading with probiotics. For the first time, we propose adding an enzymatic extract of feces to an artificial colon fluid, which mimics the total hydrolytic activity of the intestinal microbiota. The beads can be digested by fecalase with cellulase activity, indicating intestinal release. The encapsulation of LGG significantly enhanced their viability under simulated GI conditions. However, the beads, in combination with the prebiotic, provided greater protection of bacteria, enhancing their survival and even increasing cell numbers in the capsules. These data suggest the promising prospects of coencapsulation as an innovative delivery method based on the inclusion of probiotic bacteria in a symbiotic matrix.

## 1. Introduction

Microbial polysaccharides are becoming more and more attractive materials intended for use in encapsulation technology: the inclusion of biologically active substances, enzymes, vitamins, and probiotics in the matrix [1,2].

Probiotics are usually used in the form of biologically-active food additives, or even therapeutic drugs in the form of tablets, capsules, powders, and sachets. However, most often, probiotics are used as part of functional foods. Both these and other forms are applied orally and therefore enter the gastrointestinal (GI) tract, which they need to pass through safely to reach the colon where they function. The most critical points on this path are, first of all, the stomach, which has extreme pH values, and the upper parts of the intestine, where bile acids and digestive enzymes are present [3]. From this point of view, the microcapsule shell, serving as a physical barrier, protects the cells included in it and is therefore an excellent means of delivering probiotics to the lower intestine [4,5]. Hence, encapsulation ensures the viability of probiotics during their transportation to the place of action in the human body without negatively affecting their physiological properties.

On the other hand, even inside the capsule, there is not always a high viability of bacteria, which is one of the reasons for the low success of such protection of probiotics [6]. In this regard, in recent years, coencapsulation of a probiotic with a prebiotic has been proposed as an additional method, leading to an increase in the survival of probiotics in capsules [7]. A prebiotic is defined as “a substrate that is selectively utilized by host microorganisms, conferring a health benefit” [8]. The combination of a probiotic and a prebiotic is called a synbiotic, and can have either non-specific or selective effects. In the first case, a prebiotic is selected to enhance the local beneficial microbiota, i.e., it is a “universal agent”. In the second case, the prebiotic is selected specifically to support the growth of the selected probiotic, regardless of the beneficial effect on the population of other bacteria. This particular method is used in the present study.

The model probiotic *Lactobacillus rhamnosus* GG (LGG) is one of the most extensively studied lactobacilli strains with established positive effects on human health [9].

The second component of the developed system, which this study proposes to introduce into the bead together with LGG, is a selective prebiotic. Evidence suggests that such a “nutrient component” may be polysaccharide pullulan (PUL), a neutral linear polysaccharide synthesized by *Aureobasidium pullulans*, consisting of *α*-1,6-linked maltotriose residues. Since PUL has several hydroxyl groups, if necessary, it can be easily modified. PUL is able not only to stimulate the growth of these bacteria in culture experiments but also increase their viability during encapsulation [10,11,12,13].

It should also be mentioned that according to some data, prebiotics can be destroyed to a certain extent in the small intestine [14]. Hence, prebiotics also need to be protected. With this in mind, the first objective of this study was to coat the beads in a durable biopolymer that is not directly subjected to enzymatic and physical destruction in the upper parts of the digestive system. For this purpose, bacterial cellulose (BC), an indigestible polysaccharide, also a product of microbial synthesis, was used. Since only cellulolytic bacteria live in the colon, cellulose and its derivatives can be decomposed there [15].

The second objective was to determine the protective properties of beads with prebiotic PUL coated with a cellulose shell as the proposed delivery system.

Human models are ideal for determining the functional effectiveness of beads; however, there are ethical limitations associated with this method. The closest to “intestinal nature” is an in vitro dynamic model that more accurately simulates the sequential kinetic conditions in the GI tract, including the active microbiota. To simulate the conditions of the cecum, Maathuis et al. [16] added fecal samples, which were used as an inoculum in a fed-batch fermenter. In our opinion, this is a rather complex system that requires hardware design. We suggested that a simplified modification of this system could be created, in which no intestinal microbes are added to the “intestinal fluid”, except an enzymatic extract of feces. This is called “fecalase” and represents the enzymatic activity of intestinal microorganisms [17]. The results of these studies can provide predictive tools for determining the effectiveness of probiotic delivery systems in their ecological niche.

The purpose of this study was to create a symbiotic biopolymer capsule system, Alginate-Pullulan/Bacterial cellulose (Alg-PUL/BC), for targeted probiotic delivery.

## 2. Materials and Methods

### 2.1. Bacteria and Growth Conditions

*Lactobacillus rhamnosus* GG (ATCC^®^ 53103^TM^) strain was purchased from American Type Culture Collection. LGG was incubated in MRS medium (HiMedia, Mumbai, India) at 37 °C for 48 h to obtain a cell concentration of 10^10^ CFU/mL. Then, the cells were collected by a laboratory centrifuge RS-6MC (Dastan, Bishkek, Kyrgyzstan) (at 6000× *g*, for 15 min) and washed twice with saline solution.

### 2.2. Preparation of BC

*Komagataeibacter xylinus* C3 strain was isolated at the Biotechnology department, Al-Farabi Kazakh National University; the strain was deposited in the Republic Collection of Microorganisms (Astana, Kazakhstan), with the Gen Bank accession number: KU598766. Inoculum of *K. xylinus* (1%, *v*/*v*) was added to the flasks with Hestrin–Shramm broth medium (Hi-Media, Mumbai, India) and incubated statically at 30 °C for 7 days. The cultivated films were first purified by washing with deionized water, treating with 1% (*w*/*v*) NaOH at 35 °C for 24 h to remove microbial cells, and rinsed again with deionized water. The obtained films were dissolved in an aqueous solution of NaOH–urea–H_2_O (*w*/*v*: 7%–12%–83%) at a low temperature (−12 °C) for 2 min.

### 2.3. Probiotics Encapsulation

Then, 10 mL of LGG cell culture (≈10^9^–10^10^ CFU/mL) was carefully mixed with 40 mL of a granule-forming suspension of 2% sodium alginate (Sigma-Aldrich, Taufkirchen, Germany) (alginic acid sodium salt from brown algae, guluronic acid or glucuronic content ∼65–70%; mannuronic acid content ∼5–35%) solution. Beads were prepared by the extrusion method from a 10 mL plastic syringe through a 20-gauge needle (0.9 mm diameter) into a beaker, at a distance of 25 cm, containing calcium chloride (Fisher Scientific Inc., Ottawa, ON, Canada) solution (1%, *w*/*v*) with gentle agitation at room temperature. Armed MP-2003 syringe dispenser (Shanghai Leien Medical Equipment Co. Ltd., Shanghai, China) was used for extrusion. The formed beads were left to harden for 30 min and then washed with sterile distilled water.

To obtain Alg/BC samples, Alg beads were covered with an additional layer of 0.5–2% BC solution and incubated for 30–40 min on an orbital shaker incubator ES-20 (Biosan, Riga, Latvia) (130 rpm).

To obtain hybrid beads of PUL (Hayashibara Biochemical Laboratories, Okayama, Japan) with BC, a mixture of Alg (2% solution) and PUL (1–2% solution) was first prepared in a ratio of 1:1. 10 mL of probiotic culture was added to the resulting mixture at a cell concentration of at least 10^10^ CFU/mL. The mixture was then stirred on a homogenizer DG-360 (Stegler, Shanghai, China) at 3000 rpm for 30 min. The resulting mixture was passed through a syringe needle into a sterile calcium chloride solution (1%, *w*/*v*). After, the beads were kept in a solution of calcium chloride for 30 min for immobilization and washed twice with distilled water. The obtained beads Alg + PUL were put into a 0.5% BC solution, incubated on a shaker, and washed twice with sterile distilled water. The capsules were stored in sterile vials at 4 °C and used in further experiments.

### 2.4. Scanning Electron Microscopy (SEM)

SEM micrographs were obtained using a scanning electron microscope (Quanta 3D 200i, Hillsboro, OR, USA). To obtain samples, the beads were freeze-dried on a Lyoquest-80 lyophilizer (Telstar, Madrid, Spain). Dried samples were placed on strips of double-sided carbon tape attached to aluminum loops. SEM of all samples was carried out at the following parameters: accelerating voltage 15 kV; working distance ≈10 mm. All measurements were carried out in a high vacuum mode of 10^−3^ Pa.

### 2.5. Mechanical Characterization

The Instron bursting machine (model 3365, Norwood, MA, USA) was used to determine the tensile strength (MPa) in uniaxial mode. Randomly selected beads were placed on the lower platform of the bursting machine and subjected to pressure in the vertical direction from top to bottom until the bead was destroyed. The upper platform with a flat tip was connected to a force sensor, which, during compression, recorded the force values acting on the beads. The mechanical properties of each sample were the average values determined from fifty specimens.

### 2.6. Encapsulation Efficiency

The capsules containing LGG bacteria were decapsulated using cellulase from *Trichoderma* sp. (Sigma-Aldrich, Taufkirchen, Germany). Cellulase solution was made by dissolving 50 mg/mL enzyme in deionized water. The samples were added to cellulase solution (1:10), followed by shaking at 37 °C until bacteria were released from beads completely. The viability of released cells was determined by plating serial dilutions of the resulting suspension on MRS agar medium (Hi-Media, Mumbai, India). Colony-forming units were counted after 72 h of incubation at 37 °C.

The encapsulation efficiency (E_e_) was determined by the equation:

(1) $$\mathrm{Ee}=\frac{N\times M}{N0}\times 100$$

where N is the number of viable cells released from 1 g of beads, M is the total mass of the collected beads, and N_0_ is the number of free cells before treatment.

### 2.7. Survival of LGG in Simulated Gastric Fluid (SGF) and Simulated Duodenum Fluid (SDF)

SGF was prepared by dissolving pepsin in sodium chloride solution (0.2%, *w*/*v*) to a final concentration of 3 g/L, and pH was adjusted to 2 with hydrochloric acid. SDF was prepared by dissolving pancreatin in sodium chloride solution (0.2%, *w*/*v*) to a final concentration of 1 g/L, with 4.5 g/L bile salts, and pH was adjusted to 6.8 with sodium hydroxide. Both solutions were filtered for sterilization through a 0.45 μm membrane. All reagents were purchased from Veld (Almaty, Kazakhstan).

The encapsulated samples (1 g) were placed in 10 mL of SGF. The tubes were incubated on an orbital shaker incubator ES-20 (Biosan, Riga, Latvia) (150 rpm) at 37 °C for 1–2 h. The samples were collected after 2 h in SGF, transferred into 10 mL of SDF, and incubated as described above for SGF.

At the end of the incubation period, each sample (1 g) was removed and rinsed with distilled water. The beads containing probiotic bacteria were disintegrated using cellulase. Surviving bacteria were enumerated by pour plate counts in MRS agar incubated at 37 °C for 72 h. The survival of probiotic LGG was presented as a number of viable cells (log CFU/g). The following equation was used to calculate the survival rate % of encapsulated bacteria cells.

(2) $$\mathrm{Survival}\;\mathrm{rate}\;\%\;=\frac{\log\;\mathrm{CFU}/g\;\mathrm{after}\;\mathrm{treatment}\;}{\log\;\mathrm{CFU}/g\;\mathrm{before}\;\mathrm{treatment}}\times 100$$

For the free cells, 1 mL of LGG suspension was inoculated into 9 mL of SGF. After incubation, 1 mL of suspension was collected and transferred into 9 mL of SDF solution. The incubation conditions for free bacteria were the same as for beads. At the end of the incubation period, the survival of free cells was determined in the way described above.

### 2.8. Preparation of Enzymatic Fecal Extracts (Fecalase) and Release of LGG into Simulated Colon Fluid (SCF)

Enzymatic fecal extracts were prepared from feces collected from three healthy donors on a regular diet (two females and one male, aged 22–30 years) who did not take pro- or prebiotics and antibiotics for at least 3 months before fecal sample donation. The fecal samples were suspended in 1 mL potassium phosphate buffer (0.01 M, pH 7.4) and homogenized for 1.5 min using a Mini-Beadbeater (BioSpec, Bartlesville, OK, USA). The suspension was centrifuged at 2000× *g* for 5 min using a Minispin centrifuge (Eppendorf, Hamburg, Germany), followed by another centrifugation of supernatant at 10,000× *g* for 20 min. The supernatant (fecalase) extracted after the second centrifugation was filtered for sterilization through a 0.45 μm membrane and used for the assay due to its ability to break down the cellulose shell of the capsules and release its content into SCF.

After 2 h incubation in SDF, beads (1 g) were placed in 9 mL of SCF (0.2 g/L of potassium chloride, 8 g/L of sodium chloride, 0.24 g/L of potassium phosphate monobasic, 1.44 g/L of sodium phosphate dibasic, pH 7.2). Then, 1 mL of fecalase was added to this solution. The free bacteria were treated similarly. The tubes were incubated on an orbital shaker incubator ES-20 (150 rpm) at 37 °C for 3–18 h. At the end of the incubation period, a 1 mL aliquot was removed and released bacteria were enumerated by pour plate counts in MRS agar incubated at 37 °C for 72 h. The following equation was used to calculate the release rate % of bacteria cells.

(3) $$\mathrm{Release}\;\mathrm{rate}\;\%\;=\frac{\log\;\mathrm{CFU}/g\;\mathrm{of}\;\mathrm{released}\;\mathrm{viable}\;\mathrm{cells}\;}{\log\;\mathrm{CFU}/g\;\mathrm{before}\;\mathrm{treatment}}\times 100$$

### 2.9. Statistical Analysis

All analyses were conducted in triplicate and the results were presented as mean ± standard deviation unless otherwise stated. Data were analyzed using a one-way analysis of variance (ANOVA) with the Tukey test. Statistical analyses were performed using SPSS software (version 28.0, IBM Corp., Armonk, NY, USA). Significance was defined as *p <* 0.05.

## 3. Results and Discussion

### 3.1. Obtaining and Characterization of Probiotic Beads

Methods for encapsulating probiotics are very diverse and these techniques are well described in several reviews [18,19]. The preference for one or another method depends on the purpose of the study or the direction of application of the beads. The choice of the extrusion method for capsules obtaining was due to the fact that it does not require special expensive equipment, high temperatures, and the encapsulation efficiency is very high [20]. This method consists of mixing a suspension of bacteria and a hydrocolloid solution. Although PUL belongs to hydrocolloids, it is not capable of forming a gel [21], but it can be added to a solution of sodium alginate to obtain hybrid beads by the subsequent extrusion of a gel-forming agent (calcium chloride) through a nozzle into a solution. The principle of using calcium chloride as a carrier is due to the fact that Ca^2+^ forms a cross-link with guluronic acid, which is part of the Alg molecule, forming a G-G block. At the same time, the “Egg-box” model gelation mechanism is activated; guluronic acid multimers are “fixed” by ions, forming a pocket that balances negatively charged polymer chains [22].

Alg hydrogel can be called the “gold standard” for the encapsulation of probiotics since it has mucoadhesive properties and imitates the polysaccharide matrix of enterocytes, as well as the matrix of bacterial biofilms formed in the intestine by resident bacteria [23]. However, “simple” Alg capsules also have disadvantages: easy disintegration in an acidic environment, under the action of chelating agents, monovalent ions, as well as too high porosity, which can lead to rapid release of the active principle from the Alg gel [24].

To strengthen the Alg matrix, capsules are coated with additional and sometimes several layers (layer-by-layer) of other natural polymers; chitosan, gelatin, pectin, starch, and cellulose is also among them [13].

**Figure 1:**
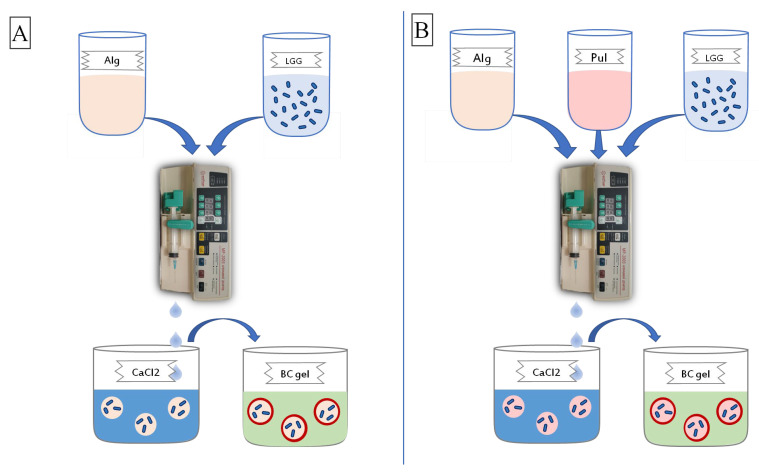
The scheme of beads with *L. rhamnosus* GG (LGG): (**A**) Alginate/Bacterial cellulose (Alg/BC), (**B**) Alginate-Pullulan/Bacterial cellulose (Alg-PUL/BC).

The design of the experiment for obtaining beads is shown in Figure 1.

The probiotic was included either in Alg alone or in Alg with PUL. Both versions of the capsules were placed from the gelling agent solution into regenerated cellulose gel to form an outer shell on the surface of the capsules.

Different ratios of polymers used may affect the properties of beads. The selection of the optimal composition was evaluated according to the parameters presented in Table 1, the key of which is mechanical strength. Since in a number of studies, the concentration of 2% Alg [25,26,27] is considered optimal, it was used for obtaining capsules by extrusion. The concentrations of PUL and BC varied in the range of 0.5–2%.

The size of the capsules has an important influence on the viability of probiotics and the sensory impact on food. There is an opinion that beads in the size range of 2000–5000 µm provide an optimal balance between these two requirements [28]. In our study, the bead size falls within this optimal range—3401 µm with PUL and 2820 µm without (Table 1). Due to the presence of PUL, the viscosity of the internal phase of the primary emulsion can increase, causing resistance to break down into smaller droplets and leading to an increase in the size of capsules [11]. Nevertheless, the size and diameter of the capsules almost coincided, i.e., no statistically significant differences (*p* > 0.05) were found between these parameters.

**Figure 2:**
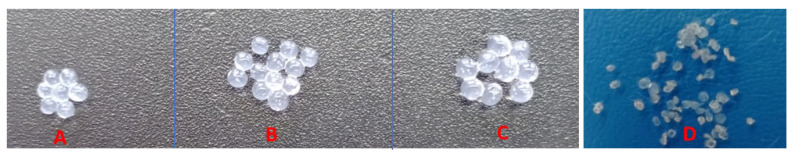
The appearance of beads: Alg (**A**), Alg/BC (**B**), Alg-PUL/BC (**C**), and dehydrated (**D**) beads.

The native beads obtained are translucent white spheres, and the dehydrated ones are “crumpled” irregularly shaped particles. In appearance, both types of capsules almost did not differ (Figure 2).

Cellulose-coated Alg capsules have a higher mechanical strength. The improvement of compressive strength is associated with good interfacial interaction between cellulose and the matrix of Alg beads due to the structural similarity of these polysaccharides [29,30]. This contributes to the formation of multiple hydrogen bonds at the interface between BC and Alg, which leads to an increase in the strength of Alg capsules coated with cellulose [25].

However, capsules with PUL are even more rigid and strong. This may be due to the filler PUL reinforcing the hydrogen network and filling the voids [31,32]. Strength is a positive technological property, since fragile capsules are easily broken and destroyed, creating problems during handling, storage, and further processing.

The E_e_ is one of the most important parameters showing the effect of the encapsulation method, as well as the matrix of the core and the capsule wall on the “quantitative loading” of bacterial cells into it. The titer of cells in Alg/BC and Alg-PUL/BC capsules reaches 10^9^ CFU/g. This is a fairly high indicator for capsules obtained by extrusion [33]. The high E_e_ obtained in our work indicates that the encapsulation process was adequate, and the wall materials were compatible with the probiotic strain.

Thus, the optimal composition of Alg-based capsules: 2% Alg + 2% PUL, 2% Alg + 0.5% BC, and 2% Alg + 2% PUL + 0.5% BC (Table 1).

**Figure 3:**
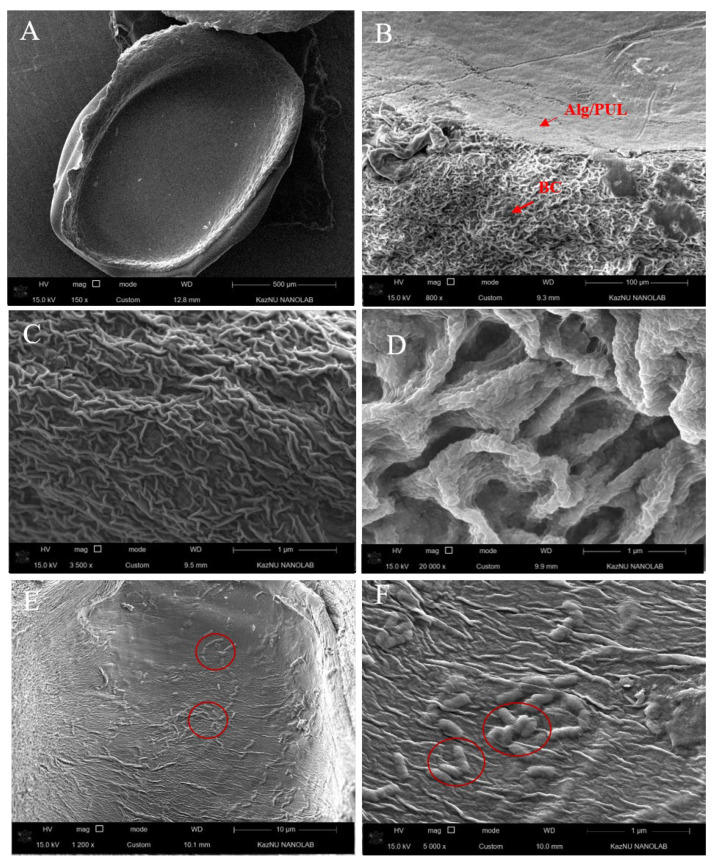
SEM images of Alg-PUL/BC beads: in cross section (**A**), in the shell section (**B**), bead shell (**C**), BC pores on the shell (**D**), LGG cells inside the bead (**E**,**F**).

The SEM study (Figure 3) demonstrated that bacteria was included in the matrix of the capsules (in the core).

The cellulose shell is also clearly visible. Moreover, it looks folded and dense and has a multi-layered structure covering the core. The formation of such a dense shell is associated with a unique three-dimensional BC network. The pore size is in a range that is insufficient for the release of encapsulated probiotics into the environment. Therefore, such a shell should be a strong framework to protect probiotics when passing through the digestive system. An experiment confirming this is presented in the next section.

### 3.2. *Survival of Free and Encapsulated Bacteria in SGF and SDF*

Protecting probiotic cells from exposure to a low pH gastric environment, bile salts, and hydrolytic enzymes is one of the primary objectives of encapsulation. Although the ultimate model for determining the functional effectiveness of capsules is a human organism, this “model” has ethical limitations. Therefore, in most such studies, an “artificial GI tract” system is used, simulating the physicochemical conditions of the main parts of the digestive system: stomach and the small and large intestines [11,12,31,34,35]. This is usually a buffer in which the pH value characteristic of a particular department is maintained and various digestive enzymes are added. In these departments, free and encapsulated cells are kept for a certain time, after which their number is determined.

**Figure 4:**
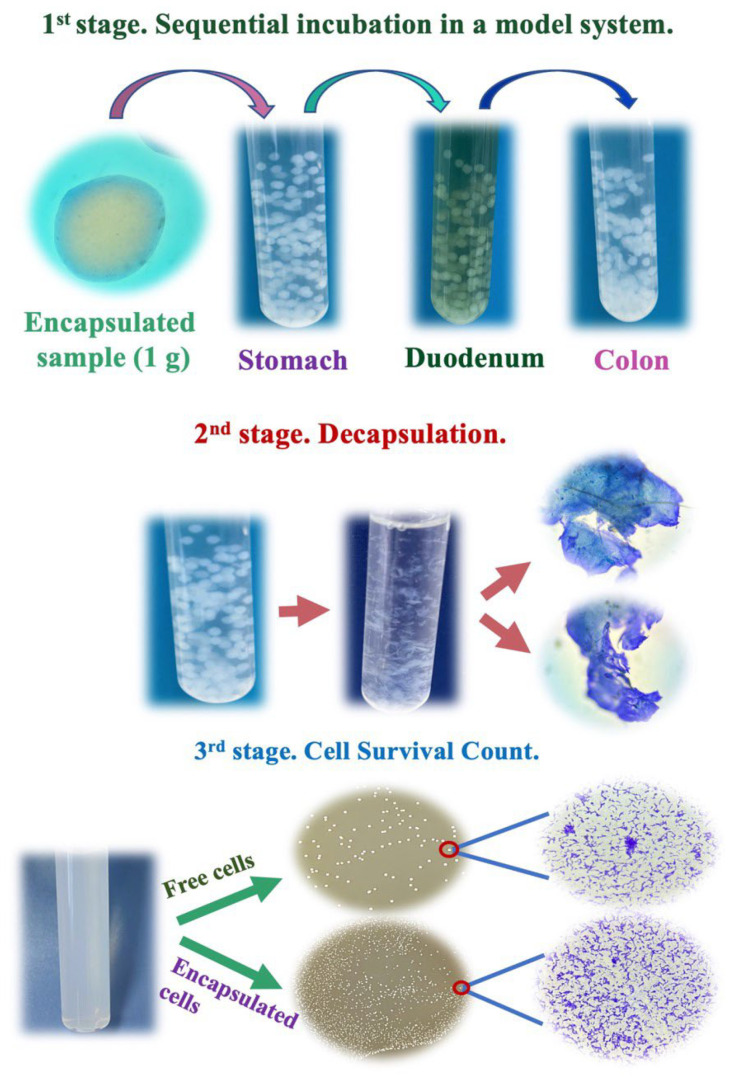
Experimental design of the simulating gastrointestinal (GI) tract.

To determine the effect of encapsulation, studies were conducted on the comparative survival of free and encapsulated LGG cells in SGF, SDF, and SCF, i.e., in the in vitro system [36]. In general, the design of this series of experiments is shown in Figure 4.

In the 1st stage, the encapsulated cells were sequentially incubated for 2 h in SGF, SDF, and 18 h in SCF. The reason for this is to simulate the condition of the GI tract. To determine cell survival count after sequential incubation, LGG-loaded capsules were submitted to the decapsulation for cell survival count (2nd stage). According to some reports, capsules have been decapsulated using citrate and phosphate buffers [37]. In studies, where cellulose was a part of the encapsulation system, peptone water solution, phosphate buffer, and mixing were used to decapsulate beads [31,34,35]. In our research, capsules have not been able to disintegrate in these solutions. This could be due to the difference in the cellulose used. The fibrous structure of BC provides excellent mechanical properties [38], which probably made it impossible for capsules based on BC to disintegrate in citrate and phosphate solutions. Cellulose is mainly degraded by the cellulase enzyme [39]. Thus, in our study, to decapsulate the cellulose capsule shell, cellulase was used, followed by enumeration by pour plate counts in a nutrient medium (3rd stage).

**Figure 5:**
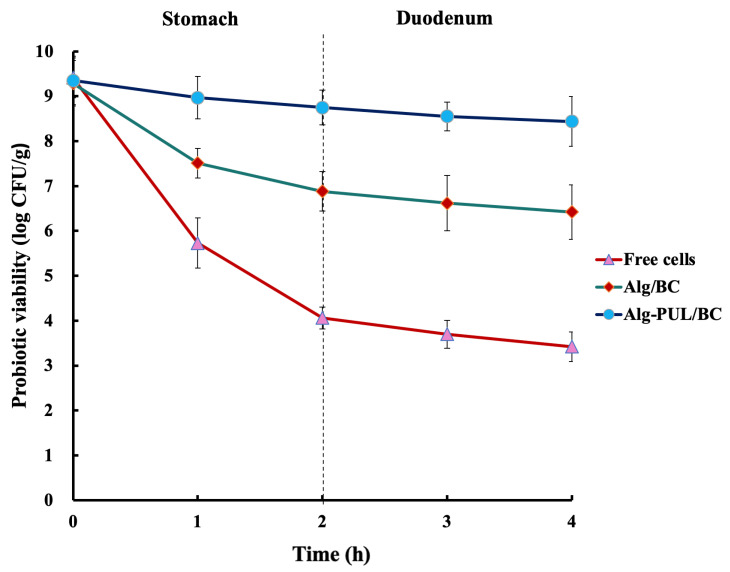
Survival of free and encapsulated LGG in upper GI conditions.

The effect of capsule coating on LGG protection against SGF was studied by comparing the viability of free and coated cells over 1–2 h. They were incubated in SGF for 2 h because food is usually in the stomach for this period [40]. The viability of free and coated cells in SGF is shown in Figure 5.

There was a noticeable decline in the viability of free probiotic cells in SGF at pH 2. The viable cell count of free cells dropped sharply in the first hour and continued to decline by 5.3 log units with a 43% survival rate. This is in agreement with other studies showing that LGG is acid sensitive [41,42,43,44,45].

Encapsulation of LGG into Alg/BC and Alg-PUL/BC beads offered more significant protection (*p <* 0.05). The viability of bacteria in beads based on BC exopolysaccharide was reduced by 2.41 log units, which provides a 74% survival rate. However, using PUL/BC as coating materials enables higher protection against low pH: 94% of cells in such capsules remained alive.

Studies where cellulose was a part of the encapsulation system demonstrated that the survivability of bacteria cells was around 83–91% [31,34]. A decrease in cell titer after incubation in SGF was also observed in capsules made of other polymers such as soy protein isolate, poly-L-lysine, or isomalto-oligosaccharide [45,46].

By comparing the loss of bacterial viability in the presence of BC and PUL/BC, it was found that the bacteria encapsulated with the prebiotic had a greater protective effect on bacteria against acidic conditions in SGF. This is in agreement with the findings reported by Çabuk et al. [11]. Their results demonstrated that probiotic cells were better protected in the presence of combined wall material PUL/whey protein after exposure to stomach conditions. PUL’s ability to block the pores in the BC network led to the protective effect of the capsule. The large pores within a gel network led to a rapid release of encapsulated bioactive substances within the GI tract [47,48]. A similar effect was observed by Iyer et al. [49], who revealed that starch granules entrapped inside a Ca-alginate matrix might stop acid diffusion into the capsules.

The next stage of the GI tract, in which there are also bacteria-damaging factors (bile salts, hydrolytic enzymes of the pancreas), is the duodenum [3]. Capsules were extracted from SGF and transferred into SDF, followed by further incubation (Figure 2).

The viable cell counts for free cells dropped by 0.64 log CFU/g after 2 h incubation. According to other results, *L. rhamnosus* demonstrated strong bile resistance [50] and its viability decreased by approximately 0.5 log CFU/mL after 90 min of exposure to ox gall [51]. In contrast, Karu et al. [52] reported that the viability of LGG had reduced by about 4 and 5 log units after treatment with bile.

In the case of Alg/BC-coated capsules, the number of probiotic cells fell from 6.88 to 6.42, which was not statistically significant (*p* > 0.05). There was only a slight decrease in the viability of cells with Alg-PUL/BC coating by 0.31 log CFU/g after SDF incubation. The probiotic survival decreased proportionally by the time the cells were subjected to SGF and SDF solutions, which was in agreement with Morsy et al. [53], in which Alg 2%  +  anthocyanin 0.1%  +  whey protein 2%  +  PUL 2%  +  cocoa butter 1% were used as the encapsulation materials of LGG. Encapsulated bacteria survival level in Alg/BC and Alg-PUL/BC coatings after exposure to SDF for 2 h was 69% and 90%, respectively. In comparison, in a study by Afzaal et al. [34], where cellulose and chitosan were used as wall materials to encapsulate *L. plantarum*, the cell survivability reached 86% after exposure to bile conditions.

Alg-PUL/BC demonstrated effective protection against the damage of the bile salt solution. This can be explained by the reduced porosity and thicker structure that a double layer offers, which can prevent bile from entering the blended network [54]. According to Youssef et al. [55], the viability of *L. salivarius* encapsulated in Alg and coated with carboxymethyl cellulose was higher than the probiotics encapsulated in Alg alone under thermal treatment, storage, and simulated GI conditions. The combination of BC with prebiotic PUL not only increased the viability of probiotic bacteria but facilitated the formation of the integrated structure of beads. Capsules with larger size generally would not undergo the same rate of matrix degradation as the smaller capsules resulting in a longer release profile because they have a lower surface area to volume ratio [56,57]. According to these findings, Alg-PUL/BC beads retained more effectively under upper GI tract conditions. As a result, it was expected that the beads would reach the colon where they can exhibit their beneficial properties.

### 3.3. Release of LGG into SCF

In the traditional in vitro system simulating the colon conditions (pH 7.2 + electrolytes), cellulose-coated capsules were almost not destroyed, since, as has already been shown, cellulase treatment was needed for their destruction. It is well known that this enzyme is traditionally present in special sections of the GI tract of ruminants and other animals that feed exclusively on plant foods [58]. However, cellulolytic microorganisms have been found in human feces, in which all components of the microbial intestinal biofilm are present [59]. In this regard, it was hypothesized that cellulase should be present in the contents of the colon, namely in the enzyme fraction of feces: fecalase.

To detect cellulase activity, fecalase was placed in wells cut on agar with the substrate 1% carboxymethyl cellulose, which was then spilled with Congo red or Lugol solutions [60]. The presence of the enzyme was recorded by the appearance of hydrolysis zones, the diameter of which averaged 23–26 mm, which indicated significant cellulase activity (data not provided).

**Figure 6:**
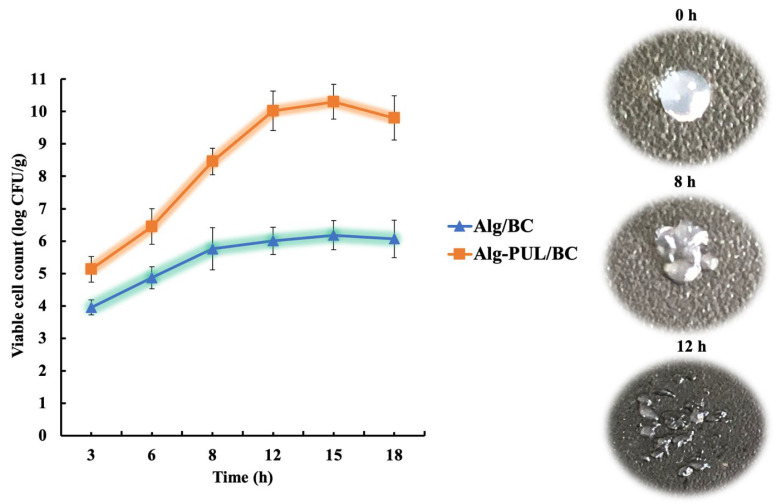
LGG release profile from beads into simulated colon fluid (SCF). All the samples were sequentially immersed in simulated gastric fluid (SGF) and simulated duodenum fluid (SDF) for 4 h before transferring into SCF.

For the assay of the determination of encapsulated LGG release, beads were transferred into SCF with fecalase at pH 7.2 and incubated for a further 18 h. The results are shown in Figure 6.

Results indicated that cell counts of released bacteria increased with incubation exposure time. The surface of the capsules initially swells, then their porosity increases [61]. This leads to the release of bacteria from the core of capsules, the cellulose shell of which is gradually destroyed as the enzyme was exposed. Then, 60% (3.95 log CFU/g) of encapsulated probiotic bacteria from Alg/BC capsules were released after the first 3 h of incubation in SCF, and within 15 h this process was completed, reaching viable cell numbers of 6.18 log CFU/g. Then, the curve reached a plateau. The final number of viable cells released from the BC beads was 0.24 log CFU/g lower than the number after exposure to duodenum conditions (6.42 log CFU/g). The total loss of probiotic bacteria in Alg/BC beads by the end of the simulated GI conditions was 3.11 log CFU/g, while in the free state, only 10^3^ CFU/mL cells reached the colon, i.e., the loss was 6 log units.

Under the same experimental conditions (SCF + fecalase), after 15 h, the highest number of viable bacteria released from Alg-PUL/BC capsules was recorded: 10.3 log CFU/g. It turned out that the number of cells in these capsules that reached the “terminal station” not only did not decrease but even increased by a log unit compared to the initial value (9.35 log CFU/g).

This phenomenon indicates that LGG in such a “container” not only survived under the harsh conditions of the human GI tract but also retained its ability to produce biomass inside the capsule. In its core, bacteria are distributed in the matrix of Alg and PUL, which is an effective prebiotic, i.e., a source of selective nutrition for this strain. Thus, coencapsulation of a probiotic with a suitable prebiotic and further coating with cellulose significantly increased the survival of bacteria compared to the delivery system without a prebiotic.

## 4. Conclusions

This study not only confirms that Alg-PUL/BC capsules can be used as a protective carrier for probiotic bacteria but also demonstrates the prebiotic effect of adding PUL, which leads to the reproduction of bacteria inside the capsule during intestinal transit. The intestinal microbiota contains microorganisms degrading cellulose. The combination of the peristaltic movement and the low amounts of cellulase presented in the lower GI tract is the result of bacteria release. In the presence of gut enzymes, the coating material was digested, resulting in the BC network’s collapse. The degradation of the network led to the release of probiotic cells from the core. Delivery of the probiotic together with a nutrient source, providing its high cell number, will allow it to compete with the local microbiota, and, as a result, to colonize the colon, benefiting the host. Further research is needed on the selection of bacterial strains and carrier matrices, as well as the development of appropriate technologies that promote the survival of bacterial cells under other types of stress (heating, freezing, osmotic and oxygen stress, drying, and storage).

## Figures and Tables

**Table 1:** Encapsulation efficiency (E_e_), size, and mechanical strength of probiotic beads.

Type	Concentration, g/100 mL		E_e_, %	Size, µm		Mechanical Strength, MPa
	PUL	BC		Dry	Wet	
Alg, 2%	-	-	78.8 ± 3.88	801 ± 55.1	2550 ± 127.1	26.6 ± 0.62
Alg, 2% + BC	-	0.5	81.2 ± 4.01	897 ± 60.1	2820 ± 143.0	28.8 ± 0.83 *
	-	1	80.2 ± 4.13	878 ± 59.9	2819 ± 140.1	26.9 ± 0.63
	-	2	77.2 ± 3.89	871 ± 71.4	2815 ± 168.9	24.9 ± 0.55
Alg, 2% + PUL/BC	1	0.5	88.3 ± 4.41 *	908 ± 61.1 *	3341 ± 233.4 *	36.8 ± 0.58 *
	1	1	87.6 ± 4.33 *	903 ± 72.7 *	3367 ± 167.1 *	35.2 ± 0.68 *
	1	2	87.1 ± 4.36 *	887 ± 97.3	3371 ± 235.9 *	35.8 ± 0.49 *
	2	0.5	89.1 ± 4.47 *	921 ± 61.0 *	3401 ± 204.0 *	37.1 ± 0.77 *
	2	1	87.8 ± 4.39 *	910 ± 85.4 *	3351 ± 134.4 *	34.1 ± 0.73 *
	2	2	87.6 ± 4.33 *	820 ± 61.3	3373 ± 168.1 *	34.6 ± 0.69 *

Alg—alginate, BC—bacterial cellulose, PUL—pullulan; * differences between alginate and hybrid beads were significant (*p* < 0.05).

## Data Availability

Data are contained within the article.
